# Drug-related admissions and hospital-acquired adverse drug events in Germany: a longitudinal analysis from 2003 to 2007 of ICD-10-coded routine data

**DOI:** 10.1186/1472-6963-11-134

**Published:** 2011-05-29

**Authors:** Jürgen Stausberg, Joerg Hasford

**Affiliations:** 1 Institut für Medizinische Informationsverarbeitung, Biometrie und Epidemiologie (IBE), Ludwig-Maximilians-Universität München, Marchioninistraße 15, D-81377 München, Germany

## Abstract

**Background:**

Adverse reactions and medication errors are complications of drug use. Spontaneous reporting systems and pharmacoepidemiological studies incompletely detect the occurrence of these events in daily hospital care. In this study, the frequency and type of drug-related admissions and hospital-acquired adverse drug events (ADE) in Germany were assessed using routinely collected hospital data.

**Methods:**

The study was based on aggregated hospital routine data covering the period 2003 to 2007 and annually recorded as part of the further development of the German Diagnosis-Related Groups. The 505 ICD-10-codes indicating an ADE were categorized in seven groups according to their certainty. Primary diagnoses were considered as a proxy for drug-related admissions, and secondary diagnoses as a proxy for hospital-acquired ADE.

**Results:**

Among all hospital admissions, 5% were found to be at least possibly drug-induced and 0.7% very likely drug-induced. There was a significant increase in the overall rate of drug-related admissions over time (p < 0.038). Enterocolitis due to *Clostridium difficile *infection was the most frequent cause of a drug-related admission. About 4.5% of in-patients had experienced a hospital-acquired ADE. In addition, over the course of the study period, the overall frequency of hospital-acquired ADEs significantly increased (p < 0.001).

**Conclusions:**

In Germany, more than 5% of hospital episodes are either caused or complicated by an ADE. Between 2003 and 2007, there was a statistically significant increase in the overall rate and in some of the subcategories defined by the list of ICD-10-codes suspected to be indicative of an ADE. Before the use of routine data in pharmacovigilance and patient safety can be fully exploited, a further tailoring of both the ICD and the available variable set is needed.

## Background

Adverse drug events (ADE) occur as a consequence of medication errors or as adverse drug reactions (ADR). Studies and systematic reviews have revealed that 5-10% of all internal-medicine admissions result from ADRs. Moreover, 5-10% of all in-patients are expected to suffer from severe ADRs, and ADRs rate among the leading causes of death in the Western world [1-6]. However, 30-40% of those ADR are considered to be preventable [3,7]. A report by the US American Institute of Medicine, "To Err Is Human: Building a Safer Health System," together with other, similar international reports, has had a lasting effect on public awareness with regard to safe pharmacotherapy and the prevention of medication errors [8]. In addition, as a result of these publications, critical-incident reporting systems and computerized physician-order entry systems combining patient- and medication-related information were recommended and subsequently introduced as a key approach to the reduction of medication errors [9-11].

With respect to ADRs, a variety of monitoring systems have been developed, the oldest and most common one being the spontaneous reporting system [12]. In addition, there have been ad hoc epidemiological studies such as cohort and case-control studies whose aim was to elucidate the etiology of adverse events. Furthermore, the use of databases from health insurances and health care providers is very common in North America, the UK and Scandinavian countries where, compared to Germany, data confidentiality laws are less restrictive [13-15]. The traditional monitoring systems lack either in completeness due to an unsystematic approach [16] or lack in representativity due to a selective study design (cf. [17,18] as examples for studies in single hospitals). By contrast, routinely collected reimbursement data promise both, completeness and representativity, through the coverage of nearly the whole population [19]. However, routinely collected reimbursement data have only rarely been evaluated for their suitability in pharmacovigilance and, especially, in the assessment of the ADEs of in- and out-patients in England and the United States [20-23]. Nonetheless, as the results of those few studies were in part encouraging, we decided to analyze the suitability of such databases in Germany.

In a recent report focusing on data from 2006, we showed that routinely collected hospital reimbursement data can be used to identify the frequency and type of ADE [24]. The present study explores this innovative approach in a nearly complete longitudinal sample of all in-patients. Specifically, we used this database to examine the frequency of drug-related admissions in Germany and of hospital-acquired ADEs during the years 2003 to 2007.

## Methods

### Definition

An ADE was defined as an injury resulting from a medical intervention related to a drug and thus included medication errors and ADRs [25-27]. Since a clear differentiation between ADRs and medication errors was not possible based on the available routine data; both events were subsumed within the definition of an ADE. Therefore, we refer to an ADR only if this term was used in the literature, even though the literature does not always provide a clear definition of an ADR.

### Database

German hospitals are obliged to annually deliver a standard data set to the Institute for the Hospital Remuneration System (InEK). The data consist of information on all in-patients who are evaluated according to the system of diagnosis-related groups (DRG); thus, data from psychiatric and psychotherapeutic departments are excluded. The InEK uses the collected data for further development of the German DRG system (G-DRG). The hospital routine data comprise, among other items, diagnoses coded by the International Statistical Classification of Diseases and Related Health Problems, 10th Revision, German Modification (ICD-10-GM) and procedures coded according to a national classification of operations and procedures (OPS). The OPS lists only a few drugs intended for reimbursement; otherwise, medication as such is excluded.

Cleared and aggregated data are freely published as Microsoft Access files under http://www.g-drg.de/. The public data cover only those patients with a normal length of stay (LOS). In principle, "normal" is defined for each DRG as ranging between one-third of the LOS arithmetic mean and the LOS arithmetic mean plus two standard deviations. One sheet of the database shows the ICD-10-GM codes together with information on the frequency as a primary and as a secondary diagnosis for each of the years 2003 to 2006. For 2007, the InEK published this sheet only for a voluntary sample of 10% of the hospitals delivering additional data from cost-unit accounting. The variables, definitions, and data-sampling procedures of the data collections between 2003 and 2006 and during 2007 were identical.

A projection of the results to the total number of in-patients in Germany for the years 2003 to 2007 is implemented assuming the representativity of both the patients with a normal LOS and the hospital sample. The total number of in-patients was drawn from further information provided by the InEK.

### Identification of relevant ICD-10 codes

In the ICD-10-GM, 505 codes indicating a possible ADE were identified (cf. appendix for a list of the codes) [24]. This list extends previous work [11,28,29]. The identified codes were classified into seven categories, each with respect to its validity as an indicator for an ADE and its definition in the ICD-10. These categories are as follows:

• Category A.1: A drug-related causation was noted in the ICD-10, e.g., G44.4 "Drug-induced headache, not elsewhere classified."

• Category A.2: A drug- or other substance-related causation was noted in the ICD-10, e.g., I42.7 "Cardiomyopathy due to drugs and other external agents."

• Category B.1: The event was denoted as a drug poisoning, thus implying an unphysiological dosage, e.g., T36.0 "Poisoning: Penicillins."

• Category B.2: The event was denoted as poisoning by or harmful use of drugs or other substances, e.g., T50.9 "Poisoning: Other and unspecified drugs, medicaments, and biological substances."

• Category C: A drug related causation was very likely, e.g., A04.7 "Enterocolitis due to *Clostridium difficile*."

• Category D: A drug-related causation was likely, e.g., F52.2 "Failure of genital response."

• Category E: A drug-related causation was possible, e.g., J81 "Pulmonary edema."

Regarding categories A.2 and B.2, other substances or measures may have caused the adverse event. The coding of categories C to E lacks an explicit reference to a medication. The German modification of the ICD-10 offers only a few codes for "Complications of medical and surgical care" (group Y40-Y84), detailed in the chapter "External causes of morbidity and mortality." In particular, there is no information on specific drugs. These codes were assigned to categories A2 and C.

Due to repeated changes in the ICD-10-GM, the code list differs from year to year and the number of codes has increased from 482 (in 2003) to 505 (in 2009). In terms of content, the list has remained unaffected. The exception was in 2003, when the code for drug-induced agranulocytosis and neutropenia was not available in the ICD-10. Table 1 shows the number and percentage of codes for each of the seven categories. Over 70% of the codes (categories A to C) indicate an ADE as being very likely.

**Table 1:** Distribution of ICD-10-GM codes considered indicative of an adverse drug event (ADE)

Category	Definition	Number of codes	Proportion of all codes
A.1	Caused by a drug	104	20.6%
A.2	Caused by a drug or other substance	78	15.4%
B.1	Poisoning by drug	133	26.3%
B.2	Poisoning by or harmful use of a drug or other substance	15	3.0%
C	ADE very likely	30	5.9%
D	ADE likely	83	16.4%
E	ADE possible	62	12.3%

Total		505	100.0%

### Drug-related hospital admissions

After thorough examination and analysis, the diagnosis that directly led to the hospital admission was defined as the primary diagnosis, according to the German coding standards [30]. There was exactly one primary diagnosis for each hospital episode. An ADE coded as primary diagnosis was therefore always acquired before the current episode. For that reason, the results related to the primary diagnoses served as a proxy for drug-related hospital admissions.

### Hospital-acquired ADE

According to the German coding standards, every disease other than the one coded as the primary diagnosis and requiring diagnostic, therapeutic, supportive, nursing, or monitoring efforts has to be coded as a secondary diagnosis [30]. This definition therefore includes comorbidities as well as complications. A definite distinction between "prior to admission" and "acquired during hospitalization" was not possible because a "present on admission" indicator was not available [31]. However, the results related to the secondary diagnoses were used as a proxy for hospital-acquired ADEs. Between 2003 and 2006, the number of secondary diagnoses per case increased from 3.5 to 4.4 (2003: 3.5, 2004: 4.0, 2005: 4.1, 2006: 4.4, and 2007: 4.3). To control for this increase, the results for hospital-acquired ADEs were standardized based on the average number of such events in 2006, as the reference year. It should be noted that one case can simultaneously be assigned several codes from one or more of the categories A to E.

### Statistics

Data from the years 2003 to 2006 represented three-quarters of the target population of all in-patients in Germany, with both absolute and relative frequencies reported. To identify a trend within the categories, a linear regression was performed using the nationwide rate as the dependent variable and the year as the independent variable. Statistical significance was assumed at p ≤ 0.05. The data were administered with Microsoft Access 2007 and analyzed with Microsoft Excel 2007 and PASW Statistics 17.0.

## Results

**Figure 1:**
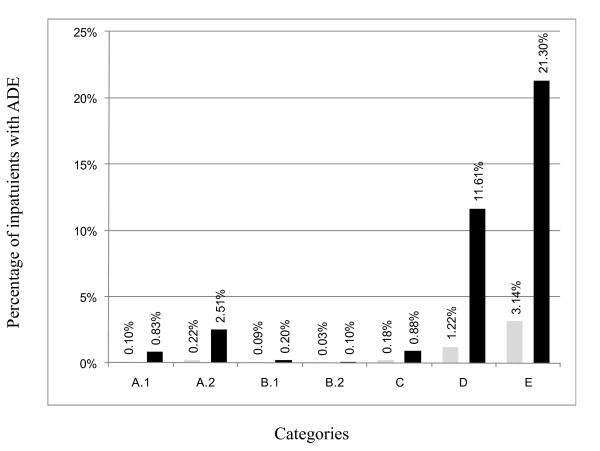
**Average annual rates of adverse drug events (ADEs) in the seven categories (left, gray column: drug-related admissions; right, black column: hospital-acquired)**.

About 48 million hospital episodes were included in this analysis of ADEs. From 2003 to 2006, the InEK sheets covered between 11,205,770 and 11,978,011 hospital episodes (Table 2), representing between 72% (2003) and 79% (2006) of all episodes recorded with the G-DRG system. The 10% sample of hospitals in 2007 consisted of 1,964,313 episodes (13% of all episodes in 2007). Figure 1 shows the averages of the annual ADE rates in the seven categories.

**Table 2:** Study population

	Year				
	**2003**	**2004**	**2005**	**2006**	**2007**^**†**^

In-patients (number)	11,912,797	11,205,770	11,269,412	11,978,011	12,244,671
PCCL (percentage)					
0	56.45	52.25	52.75	53.64	55.16
1	0.62	0.60	0.52	0.80	1.41
2	14.73	15.07	13.83	13.40	14.35
3	16.67	17.75	17.51	17.59	17.69
4	11.52	14.33	15.40	14.56	11.39
Sex (percentage)					
Male	45.40	45.33	45.65	45.95	45.96
Female	54.60	54.66	54.34	54.04	54.04
Age group (percentage)					
<18	13.38	12.97	12.60	12.08	11.88
18 to 59	39.43	37.84	37.44	36.92	36.62
60 to 79	35.61	36.45	36.69	36.93	36.86
>79	19.84	21.65	22.41	23.52	24.13
Length of stay					
Mean (days)	7.31	7.51	7.29	7.35	7.26
Stdev (days)	3.15	3.16	3.03	3.05	3.03

Characteristics of in-patients with a normal length of stay. PCCL is the patient clinical complexity level, a comorbidity measure used within the G-DRGs (0 = no complications or comorbidities, 4 = catastrophic complications or comorbidities). The figures published per DRG were weighted with the number of in-patients per DRG.

^†^The study sample for 2007 included only 1,964,313 in-patients with information on diagnoses.

### Drug-related hospital admissions

The results from the years 2003 to 2007 for the various categories are presented in Table 3. Between 0.54% and 0.67% of the episodes were assigned a primary diagnosis from categories A to C (2003: 0.54%, 2004: 0.62%, 2005: 0.63%, 2006: 0.67%, and 2007: 0.64%). These episodes were very likely caused by drug-related hospital admission. The missing code for drug-induced agranulocytosis and neutropenia accounts for the lower rate of 0.54% in 2003. Five percent of the admissions were at least possibly drug related (categories A-E). Over time, there was a highly significant rise in category C, implying an increase in very likely ADEs (p < 0.001). Likely ADEs (category D, p = 0.029) as well as the overall rate (p = 0.038) increased considerably; in contrast to the slight decrease in drug-induced or other intoxications (category B.2, p = 0.010). There were no statistically significant changes in the remaining categories.

**Table 3:** Drug-related hospital admissions per category

Category	2003		2004		2005		2006		2007		Total
	**Rate**	**Number**	**Rate**	**Number**	**Rate**	**Number**	**Rate**	**Number**	**Rate**	**Number**	**Rate**

A.1	0.06%	9546	0.10%	14728	0.10%	14867	0.12%	18287	0.11%	17664	0.10%
A.2	0.22%	36614	0.23%	34894	0.23%	34194	0.20%	30689	0.20%	31842	0.22%
B.1	0.08%	13676	0.10%	14958	0.09%	13582	0.11%	16840	0.07%	10424	0.09%
B.2	0.04%	6188	0.03%	5172	0.03%	4823	0.03%	4857	0.03%	4697	0.03%
C	0.14%	22883	0.16%	24394	0.18%	27082	0.20%	30699	0.22%	34623	0.19%
D	1.09%	181152	1.19%	179801	1.17%	175682	1.24%	187583	1.40%	218105	1.23%
E	2.95%	490194	3.10%	469040	3.16%	474140	3.36%	509535	3.10%	482905	3.14%
A - E	4.58%	760252	4.91%	742987	4.97%	744369	5.26%	798490	5.14%	800260	4.98%

Total number of in-patients		16598546		15127645		14989953		15181779		15559359	

The rate is the proportion of the sample from the respective year. The absolute number of in-patients within each category is a linear extrapolation from the sample to the target population (Germany). The total rate is the weighted average for the years 2003 to 2007.

Among categories A to C, the ten most frequent events in 2005 and 2006 were the same, with only a small change in their ranking (Table 4). In all other years, these diagnoses ranked among the top 20. The most frequent drug-related cause for a hospital admission was enterocolitis due to *Clostridium difficile *infection, responsible for almost one out of every 1000 episodes.

**Table 4:** The ten most frequent ADEs in 2006 recorded as the primary diagnosis (in descending order)

ICD-10-GM		Category	Admissions	
**Code**	**Text**		**Frequency**	**Percentage**

A04.7	Enterocolitis due to *Clostridium difficile*	C	9874	0.065
T88.7	Unspecified adverse effect of drug or medicament	A.2	5004	0.033
I95.2	Hypotension due to drugs	A.1	3720	0.025
D69.0	Allergic purpura	C	3526	0.023
T50.9	Poisoning: Other and unspecified drugs, medicaments and biological substances	B.2	3501	0.023
T78.3	Adverse effects, not elsewhere classified: angioneurotic edema	A.2	3465	0.023
L27.0	Generalized skin eruption due to drugs and medicaments	A.2	3400	0.022
K52.1	Toxic gastroenteritis and colitis	C	2727	0.018
D70.10	Drug-induced agranulocytosis and neutropenia: Critical phase <10 days	A.1	2022	0.013
T78.4	Adverse effects, not elsewhere classified: allergy, unspecified	A.2	1754	0.012

Total number of in-patients			15181779	100.000

### Hospital-acquired ADE

Between 4.46% (in 2005) and 4.60% (in 2006) of all episodes included a secondary diagnosis from categories A-C (Table 5). These diagnosis were very likely drug-induced with a suspicion for being hospital-acquired. This result represents an upper estimate because pre-admission diseases were recorded as well. There was a statistically significant increase in the number of events in categories A.1 (p = 0.008), C (p = 0.022), D (p = 0.001), E (p = 0.003), and overall (p < 0.001), and a statistically significant decrease in categories A.2 (p = 0.018), B.1 (p = 0.054), and B.2 (p = 0.005).

**Table 5:** Hospital-acquired ADEs per category

Category	2003		2004		2005		2006		2007		Total
	**Rate**	**Number**	**Rate**	**Number**	**Rate**	**Number**	**Rate**	**Number**	**Rate**	**Number**	**Rate**

A.1	0.61%	101850	0.80%	121181	0.84%	126113	0.90%	136963	1.00%	155226	0.85%
A.2	2.78%	460850	2.54%	383580	2.48%	371473	2.47%	374988	2.29%	356265	2.52%
B.1	0.24%	39127	0.23%	34158	0.21%	31197	0.21%	31816	0.14%	21425	0.21%
B.2	0.13%	20939	0.12%	17627	0.10%	15521	0.10%	14712	0.07%	11039	0.11%
C	0.79%	130548	0.81%	122058	0.83%	124617	0.92%	139172	1.05%	163740	0.89%
D	9.55%	1585917	10.79%	1632913	11.87%	1778861	12.56%	1907125	13.28%	2066457	11.74%
E	19.18%	3184349	20.36%	3080717	21.00%	3148326	22.01%	3342171	23.92%	3721954	21.40%
A - E	33.28%	5523579	35.64%	5392233	37.33%	5596108	39.17%	5946946	41.75%	6496107	37.61%

Total number of in-patients		16598546		15127645		14989953		15181779		15559359	

The rate is the proportion of the sample from the respective year. The absolute number of in-patients within each category is a linear extrapolation from the sample to the target population (Germany). Rate and number are standardized for the average number of secondary diagnoses in 2006. The total rate shows the weighted average for the years 2003 to 2007.

With minor deviations, the ten most frequent events in categories A-C in 2006 ranked among the top 20 events in other years (Table 6). The unspecific code T88.7 was the most frequent one from categories A to C in 2006 but not in 2007. In Table 6, Y57.9 is a supplementary code of the ICD-10-GM and is solely used in order to classify a disease even more precisely.

**Table 6:** The ten most frequent ADEs in 2006 recorded as secondary diagnoses (in descending order)

ICD-10-GM		Category	Admissions	
**Code**	**Text**		**Frequency**	**Percentage**

T88.7	Unspecified adverse effect of drug or medicament	A.2	83182	0.548
Y57.9	Drug or medicament, unspecified	A.2	48276	0.318
T78.4	Adverse effects, not elsewhere classified: allergy, unspecified	A.2	42677	0.281
D69.58	Secondary thrombocytopenia, not described as transfusion resistant	C	31791	0.209
A04.7	Enterocolitis due to *Clostridium difficile*	C	26341	0.174
T80.1	Vascular complications following infusion, transfusion, or therapeutic injection	A.2	20742	0.137
D70.10	Drug-induced agranulocytosis and neutropenia: Critical phase <10 days	A.1	14022	0.092
D61.10	Drug-induced aplastic anemia due to chemotherapy	A.1	13003	0.086
D69.59	Secondary thrombocytopenia, not otherwise specified	C	12357	0.081
F13.7	Mental and behavioral disorders due to use of sedatives or hypnotics, residual and late-onset psychotic disorder	A.2	12153	0.080

Total number of in-patients			15181779	100.00

## Discussion

### Drug-related hospital admissions

According to German routine data from 2003 to 2007, almost 0.7% of all hospital admissions were very likely drug-related. Reasons for admission coded as a secondary diagnosis remained unconsidered. This suggests that the results represent a lower estimate. From 2003 to 2007, there was a considerable increase in diseases that were very likely ADEs and which resulted in hospital admission. The reason for this increase is not known. The proportion of in-patients 80 years and older increased continuously between 2003 and 2007. Associated with this aging population might be an increased risk of ADEs due to multimorbidity and subsequent polymedication. Among all hospital admissions, 5% were at least possibly drug-related. Hence, a relevant number of hospital admissions in Germany can be ascribed to adverse drug reactions and medication errors. The most common ADEs remained the same over time.

In a review of 25 prospective observational studies, a 5.3% rate of ADR-related admissions was estimated [4]. Waller et al. examined drug-induced hospital admissions using the Hospital Episodes Statistics database from England [21]. Their analysis showed that 0.35% of hospital admissions were coded as 'drug-induced'. The ICD-10 codes considered by in that study roughly correspond to categories A.1 and C of the present work. The rate of 0.35% is in accordance with the range of 0.20 (2003, categories A.1 and C) to 0.34 (2007, categories A.1 and C) in Germany. The frequency of recorded ADRs has increased over time in both England and Germany. Waller et al., drawing conclusions from the Hospital Episodes Statistic in England, reported an increase of 40% between 1996 and 2000 [21], and Patel et al., in another study carried out in England, calculated an increase of 45% between 1998 and 2005 [32]. In Germany, we determined an increase of 30% in drug-related admissions between 2004 and 2007 as indicated by a primary diagnosis assigned to categories A.1 and C.

### Hospital-acquired ADE

According to our findings, an ADE as consequence of a hospital stay can be expected in almost 5% of hospital episodes. This percentage, however, has to be understood as an approximate upward assessment since there were no case-related data. At 5%, drug-related ICD codes represent almost 50% of the estimated 10% of in-patients in Germany who suffer an adverse event [33].

There has been an overall increase in hospital-acquired ADEs. A documentation artifact was controlled for in the presented study by standardizing the average number of secondary diagnoses in 2006. On the one hand, the increase in the mean number of diagnoses between 2003 and 2006 can be explained by improved completeness of recording complications and comorbidities. On the other hand, there are case-mix changes, since the population has become older. However, both decreases in the length of stay and the comorbidity measure have remained stable over time (Table 2).

The rate of suspected cases in Germany is similar to the estimate of 5.3% reported by Kongkaew et al. [4]. In Western Australia, the trend of repeated in-hospital treatments due to ADR was examined between 1980 and 2003 based on the reimbursement data of patients 60 years of age and older [28]. During the entire study period, the rate was about 5.9% but the number of repeat admissions increased. The authors exclusively used supplementary codes for the identification of an ADR according to the ICD-9 and ICD-10, such as Y57.9, and took into account both pre-admission and in-hospital ADRs [28].

The literature is not always precise in differentiating between ADEs leading to admission and hospital-acquired ADEs. For example, a nationwide study from The Netherlands analyzed primary and secondary diagnoses together to assess the frequency of ADE-related hospitalizations [34]. For 2001, the rate was 1.83%. Our results are similar to those from a retrospective review of clinical records published by Brennan et al. [35]. However, a meta-analysis of prospective studies yielded an estimate of 15.1%, which represents a considerably higher rate of ADR episodes than determined in the present study [3]. The German Coalition for Patient Safety suggests an overall estimate of 5-10% for in-hospital adverse events [33]. Most of these events occur in a surgical context, followed by ADE and systemic factors. Adverse events in connection with other diagnostic, therapeutic, or invasive procedures are reportedly less frequent [36].

### Routine data

In a few studies, only routine hospital statistics were used in the assessment of ADEs. The results are comparable to ours and it was consistently suggested that this database can contribute positively to health monitoring with respect to drug-related patient safety issues, although the issue of underassessment remains.

A differentiation between adverse drug reactions and medication errors as well as between comorbidities and complications is at the moment only partially accomplished by the ICD and the variables available in the data set. The application of routine data to medication safety and pharmacovigilance would therefore benefit from a further tailoring of the ICD-10. For example, the codes of categories A.2 and B.2 should be divided into those pertaining to drug-related events and those pertaining to other causes. This should be taken into account by the WHO in the 11th revision of the ICD. Furthermore, an enrichment of routine data by integrating data derived from other electronic sources related to hospital information systems might additionally enable an automatic signaling even on the level of the individual patient [37]. For example, lab results and sequences of lab results can deliver important information about ADEs. Also, the inclusion of a "present on admission" indicator could differentiate between complications and comorbidities [31]. However, from our point of view, this indicator only partially solves the underlying problem of distinguishing between the two conditions, as it is closely related to differing notions about comorbidity [38-40]. For example, an adverse event due to a medication error occurring in hospital stay A is still hospital-acquired if the patient is later admitted to hospital stay B, but then the event is considered "present on admission." Thus, for improved patient safety and pharmacovigilance, information is needed independently of administrative events. In the above example, the date the event was recognized should be recorded by annotating the diagnosis code, thus preserving information on the course of the disease. Medications are not currently included in hospital routine data in Germany, although some codes of the ICD-10-GM cover such information, for example in group F10-F19 "Mental and behavioral disorders due to psychoactive substance use." Thus, it might be possible to similarly correlate drugs and events for specific areas.

### Study limitations

The recording of an ADE requires the identification of a drug as the cause of the symptom or the disease. This identification may be difficult but it is imperative when using the specific codes of the ICD-10-GM (categories A.1, A.2, B.1, and B.2). In addition, differences in ADR-definitions affect adversely the reliability of ADE signal systems [41,42]. A contamination of our results due to the inclusion of other causes (e.g., self-poisoning) in categories A.2 and B.2 has to be accepted, given the mix of different causes covered by a single ICD-10-code. Furthermore, since the list of variables provided by the InEK is limited, multiple counting of a single case has to be accepted.

Reliability and validity issues of administrative data may restrict the generalization of the presented results [43]. However, the coding quality of diagnoses and procedures in German hospitals has reached a standard high enough to allow the use of hospital routine data for quality management as well as health services research [44-47]. Revenue protection claims likewise require a detailed and correct documentation.

The average number of secondary diagnoses per case increased from 2003 to 2007. To exclude changes in registration habits, we standardized the rates of hospital-acquired ADEs according to their average number in 2006.

Although routine data may well be sufficient in terms of completeness of diagnoses and operative procedures, their accuracy is not confirmed. For example, in our study, ADEs present on admission were unintentionally counted as hospital-acquired if recorded as the secondary diagnosis. We expect situations such as this one to be the exception, however, and they do not invalidate the results regarding hospital-acquired ADEs.

## Conclusions

The results of this study support the innovative approach of using hospital routine data to obtain health-related statistics regarding medication safety and pharmacovigilance. The analyses show an increase in drug-related admissions and hospital-acquired ADEs in Germany, in line with studies from other countries. This increase should be further evaluated in studies based on detailed data. Routine data provided by hospitals and physician offices are a reliable and valid platform for obtaining information on patient safety and are free of the cost resources incurred by data acquisition. Thus, a refinement of the ICD is recommended, as it will continue to improving both the monitoring of patient safety and pharmacovigilance. Moreover, the data can be used by Health Department/Ministry politicians, patient organizations, health insurance institutions, and pharmaceutical companies to evaluating drug safety over time, as a supplement to established methods such as spontaneous reporting systems. Restrictions occurring through the limited information available, such as the absence of data regarding medication, remain to be addressed.

## Competing interests

The authors declare that they have no competing interests.

## Authors' contributions

JS participated in the study design, carried out the data analysis, and drafted the manuscript. JH participated in the study design and supported the data analysis. Both authors read and approved the final manuscript.

## Appendix

List of ICD-10-GM-codes indicating a possible ADE. Codes within each category in alphabetical order.

**Category A.1**: D52.1, D59.0, D59.2, D61.10, D61.18, D61.19, D69.52, D69.53, D70.10, D70.11, D70.12, D70.18, D70.19, E06.4, E16.0, E23.1, E24.2, E27.3, E66.10, E66.11, E66.12, E66.19, G21.0, G21.1, G24.0, G25.1, G25.4, G25.6, G44.4, G62.0, G72.0, H26.3, H40.6, I95.2, J70.2, J70.3, J70.4, K85.30, K85.31, L10.5, L43.2, L56.0, L56.1, L64.0, M10.20, M10.21, M10.22, M10.23, M10.24, M10.25, M10.26, M10.27, M10.28, M10.29, M32.0, M80.40, M80.41, M80.42, M80.43, M80.44, M80.45, M80.46, M80.47, M80.48, M80.49, M81.40, M81.41, M81.42, M81.43, M81.44, M81.45, M81.46, M81.47, M81.48, M81.49, M83.50, M83.51, M83.52, M83.53, M83.54, M83.55, M83.56, M83.57, M83.58, M83.59, M87.10, M87.11, M87.12, M87.13, M87.14, M87.15, M87.16, M87.17, M87.18, M87.19, N14.0, O74.4, P04.0, P04.1, P96.2, Q86.1, Q86.2, R50.2, T88.3

**Category A.2**: D64.2, E03.2, F11.0, F11.1, F11.2, F11.3, F11.4, F11.5, F11.6, F11.7, F11.8, F11.9, F13.0, F13.1, F13.2, F13.3, F13.4, F13.5, F13.6, F13.7, F13.8, F13.9, F15.0, F15.1, F15.2, F15.3, F15.4, F15.5, F15.6, F15.7, F15.8, F15.9, F19.0, F19.1, F19.2, F19.3, F19.4, F19.5, F19.6, F19.7, F19.8, F19.9, G21.2, I42.7, L23.3, L24.4, L25.1, L27.0, L27.1, L27.8, L27.9, M34.2, N14.1, N14.2, N14.3, N14.4, O35.5, P04.4, P58.4, P93, P96.1, T78.2, T78.3, T78.4, T78.8, T78.9, T80.1, T80.2, T80.3, T80.4, T80.5, T80.6, T80.8, T80.9, T88.6, T88.7, Y57.9, Y59.9

**Category B.1**: T36.0, T36.1, T36.2, T36.3, T36.4, T36.5, T36.6, T36.7, T36.8, T36.9, T37.0, T37.1, T37.2, T37.3, T37.4, T37.5, T37.8, T37.9, T38.0, T38.1, T38.2, T38.3, T38.4, T38.5, T38.6, T38.7, T38.8, T38.9, T39.0, T39.1, T39.2, T39.3, T39.4, T39.8, T39.9, T40.0, T40.1, T40.2, T40.3, T40.4, T40.5, T40.6, T40.7, T40.8, T40.9, T41.0, T41.1, T41.2, T41.3, T41.4, T41.5, T42.0, T42.1, T42.2, T42.3, T42.4, T42.5, T42.6, T42.7, T42.8, T43.0, T43.1, T43.2, T43.3, T43.4, T43.5, T43.6, T43.8, T43.9, T44.0, T44.1, T44.2, T44.3, T44.4, T44.5, T44.6, T44.7, T44.8, T44.9, T45.0, T45.1, T45.2, T45.3, T45.4, T45.5, T45.6, T45.7, T45.8, T45.9, T46.0, T46.1, T46.2, T46.3, T46.4, T46.5, T46.6, T46.7, T46.8, T46.9, T47.0, T47.1, T47.2, T47.3, T47.4, T47.5, T47.6, T47.7, T47.8, T47.9, T48.0, T48.1, T48.2, T48.3, T48.4, T48.5, T48.6, T48.7, T49.0, T49.1, T49.2, T49.3, T49.4, T49.5, T49.6, T49.7, T49.8, T49.9, T50.0, T50.1, T50.2, T50.4, T50.6, T50.7

**Category B.2**: F55.0, F55.1, F55.2, F55.3, F55.4, F55.5, F55.6, F55.8, F55.9, T50.3, T50.5, T50.8, T50.9, T96, X49.9

**Category C**: A04.7, D69.0, D69.2, D69.57, D69.58, D69.59, E15, H91.0, K52.1, K71.0, K71.1, K71.2, K71.3, K71.4, K71.5, K71.6, K71.7, K71.8, K71.9, L51.0, L51.1, L51.20, L51.21, L51.8, L51.9, L56.2, N99.0, O74.2, O74.3, Y69

**Category D**: D62, F52.0, F52.1, F52.2, F52.3, F52.4, F52.5, F52.6, F52.7, F52.8, F52.9, H53.0, H53.1, H53.2, H53.3, H53.4, H53.5, H53.6, H53.8, H53.9, I15.81, J45.1, K25.0, K25.1, K25.2, K25.3, K25.4, K25.5, K25.6, K25.7, K25.9, K26.0, K26.1, K26.2, K26.3, K26.4, K26.5, K26.6, K26.7, K26.9, K27.0, K27.1, K27.2, K27.3, K27.4, K27.5, K27.6, K27.7, K27.9, K28.0, K28.1, K28.2, K28.3, K28.4, K28.5, K28.6, K28.7, K28.9, K29.0, L29.0, L29.1, L29.2, L29.3, L29.8, L29.9, L50.0, N17.0, N17.1, N17.2, N17.8, N17.9, N18.0, N18.80, N18.81, N18.82, N18.83, N18.84, N18.89, N18.9, N19, O26.6, O74.6, T88.5

**Category E**: E86, E87.0, E87.1, E87.2, E87.3, E87.4, E87.5, E87.6, E87.7, E87.8, I26.0, I26.9, I44.0, I44.1, I44.2, I44.3, I44.4, I44.5, I44.6, I44.7, I45.8, I47.2, I61.0, I61.1, I61.2, I61.3, I61.4, I61.5, I61.6, I61.8, I61.9, I80.0, I80.1, I80.2, I80.3, I80.8, I80.9, J38.5, J45.0, J45.8, J81, K72.0, K92.2, N62, R00.1, R06.0, R06.88, R11, R17, R21, R34, R41.0, R42, R44.0, R44.1, R44.2, R44.3, R51, R55, R58, R73.9, R74.0

## Pre-publication history

The pre-publication history for this paper can be accessed here:

http://www.biomedcentral.com/1472-6963/11/134/prepub
